# The Characterization of microRNA-Mediated Gene Regulation as Impacted by Both Target Site Location and Seed Match Type

**DOI:** 10.1371/journal.pone.0108260

**Published:** 2014-09-19

**Authors:** Wenlong Xu, Zixing Wang, Yin Liu

**Affiliations:** 1 Department of Neurobiology and Anatomy, University of Texas Health Science Center at Houston, Houston, Texas, United States of America; 2 University of Texas Graduate School of Biomedical Science, Houston, Texas, United States of America; The John Curtin School of Medical Research, Australia

## Abstract

MicroRNAs (miRNAs) are small RNA molecules that play important roles in gene regulation and translational repression. The mechanisms that facilitate miRNA target binding and recognition have been extensively studied in recent years. However, it is still not well known how the miRNA regulation is affected by the location and the flanking sequences of miRNA target sites. In this study, we systematically quantify the contribution of a wide spectrum of target sites on miRNA-mediated gene expression regulation. Our study investigates target sites located in four different gene regions, including 3' untranslated regions, coding sequences, 5′ untranslated regions and promoter regions. We have also introduced four additional non-canonical types of seed matches beyond the canonical seed matches, and included them in our study. Computational analysis of quantitative proteomic data has demonstrated that target sites located in different regions impact the miRNA-mediated repression differently but synergistically. In addition, we have shown the synergistic effects among non-canonical seed matches and canonical ones that enhance the miRNA regulatory effects. Further systematic analysis on the site accessibility near the target regions and the secondary structure of the mRNA sequences have demonstrated substantial variations among target sites of different locations and of different types of seed matches, suggesting the mRNA secondary structure could explain some of the difference in the miRNA regulatory effects impacted by these different target sites. Our study implies miRNAs might regulate their targets under different mechanisms when target sites vary in both their locations and the types of seed matches they contain.

## Introduction

MicroRNAs (miRNAs) are small approximately 18–24 nucleotide RNA molecules that play key gene-regulatory roles in plants and animals [1], [2]. These small RNA molecules exert their regulatory effects on target gene mRNAs by inducing mRNA degradation and/or inhibiting protein translation [3], [4]. They are one of the most abundant classes of gene-regulatory molecules in mammals [5], with more than two thousand and five hundred distinct miRNAs having been identified in human [6]. It has been shown that a single miRNA can modulate the expression levels of several hundreds to thousands of different mRNA transcripts [7]. However, out of the thousands of known human miRNAs, only a handful of them have been investigated for their functions experimentally [8]–[10]. The mechanisms that underlie miRNA target recognition and regulation are largely unknown despite recent studies on the identification and characterization of miRNA targets [11], [12]. It is demonstrated that a mature miRNA can guide RNA-induced silencing complex for target recognition by sequence complementarity between the miRNA and its target site in mRNAs [13], [14]. The target site often includes nucleotides that form Waston-Crick pairs with bases in the 5′ end of the mature miRNA centered on positions 2 to 7, known as the miRNA “seed region”. There are four types of target sites defined as canonical seed matches [13]. Because of this seed rule, most current experimental and computational analyses on miRNA targeting just focus on these canonical seed matches types, but numerous exceptions to the seed rule have been demonstrated [15], [16]. Many experimental studies show that non-canonical seed-matches such as those including GU wobbles or single mismatches in the target sites could also be functional and greatly affect the mRNA repression [17], [18]. However, there is lack of studies that systematically analyze and quantify the efficiency of these non-canonical seed matches in inducing translational repression of the mRNA transcripts.

In addition to the types of seed matches, another factor contributing to the versatility of miRNA regulatory effects might be the gene regions where the miRNA target sites are located. It has been shown miRNAs predominantly interact with target sites located in 3' untranslated regions (3′UTRs) of mRNA transcripts in mammals [19], [20]. However, there has been increasing evidence suggesting that target sites in coding regions (CDSs) can confer regulation but might be less effective than those in 3′UTRs on average [7], [21]. These studies analyzed miRNA target sites in CDSs independent of the existence of those in 3′UTRs, while other studies examined possible synergistic effects of target sites located in 3′UTRs and those in CDSs [11], [22], [23]. The CLIP-based experiments have demonstrated that miRNA targeting can also occur in CDSs and 5′ untranslated regions (5′UTRs) [24], [25]. In addition, a few recent studies on both prediction and experimental verification of miRNA target sites have extended the searching regions to include the 5′UTRs [26] and the promoter regions (Promoters) [27]–[29], which resulted in a more comprehensive list of miRNA targets. Although it is well known that miRNA targeting can occur in different gene regions, little is understood of how miRNAs regulate targets with the target sites located in these different regions.

Therefore, to fully understand the roles miRNAs play in regulating different biological processes, one essential step is to investigate how the interactions between miRNAs and their target genes are impacted by target sites of different seed match types and/or by target site locations in different gene regions. In this study, we have examined target sites of both canonical types as well as non-canonical types and quantified the composite effect of different miRNA seed match types in regulating protein levels. We have also investigated sequence and structural properties of target sites located in different gene regions (including 3′UTRs, CDSs, 5′UTRs and Promoters) and their impact on miRNA regulatory effects. The miRNA-mRNA pairs that we used in this study included the experimentally verified miRNA-mRNA interaction dataset from miRWalk [30], the genome wide protein-level changes following miRNA transfection [7] and the computationally predicted results in TargetS [29].

## Results

### Signal-to-noise ratio

**Figure 1:**
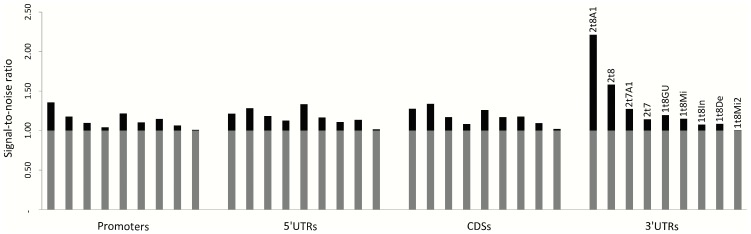
Signal-to-noise ratios of different types of seed matches in different gene regions. The signal-to-noise ratio is the ratio of the number of matches of each seed match type in the miRWalk dataset and the number of matches obtained from random shuffles of mRNA sequences.

We first used the miRWalk dataset to calculate the signal-to-noise ratios (SNRs) for nine types of seed matches located in each of the four different gene regions (the 3′UTRs, CDSs, 5′UTRs, and Promoters). Among the seed matches included in our study, there are four types of canonical seed matches: 2t8A1 (it requires Watson-Crick pairing to the 5′ region of the miRNA on nucleotides 2 to 8 and the first nucleotide of the target mRNAs being adenine), 2t8 (seed paring from position 2 to 8 in the 5′ region of the miRNA), 2t7A1 (seed paring from position 2 to 7 with position 1 of the target mRNA being adenine) and 2t7 (seed pairing from position 2 to 7). Besides these canonical seed match types, we also have studied non-canonical ones including 1t8GU, 1t8Mi, 1t8In and 1t8De (Figure S1). Type 1t8GU requires seed paring on positions 1 to 8 but allowing 1 GU wobble pair, while types 1t8Mi, 1t8In and 1t8De allow 1 bit of mismatch, insertion or deletion at the target site, respectively. The SNR for each type of seed matches located in each gene region is shown in Figure 1. The seed match type of 1t8Mi2 contains 6 bits of Watson-Crick pairing and 2 bits of mismatches from position 1 to 8 in the 5′ of miRNA, and was used as a negative control for SNR calculation. It is observed that the SNR of this seed match type is closest to 1.0 among all types of seed matches in all the four gene regions. Sequences containing this type of seed matches are very unlikely to serve as functional miRNA target sites [13], so the resulting close to 1.0 SNR value for this seed match type confirms the reliability of our SNR calculation.

In Figure 1, we can see the type of seed matches with the most significant SNR is 2t8A1, followed by 2t8. The type 2t7A1 is more significant than 1t8GU in 3′UTRs, but less significant than 1t8GU in other gene regions according to the comparison of their SNR values. The SNRs of non-canonical seed match types 1t8Mi, 1t8In and 1t8De are comparable with the canonical type 2t7. The target sites located in 3′UTRs result in the most significant SNRs, followed by those located in CDSs and 5′UTRs, whose SNR values are roughly comparable. The target sites in Promoters seem to have the lowest SNRs but still demonstrate more significant signals than the random noise.

### Impact of target sites in different gene region on protein expression levels

To compare the miRNA effects on target sites that are located in different genes regions, we analyzed the large-scale pSILAC dataset, which was obtained with five human miRNAs (hsa-miR-1, hsa-miR-16, hsa-miR-155, hsa-miR-30a and hsa-let-7b). We investigated the log2 fold changes of protein levels in response to miRNA overexpression for each of these five miRNAs. In our analysis, we first grouped all genes included in the pSILAC dataset according to the location of the target sites in their corresponding mRNA sequences. Five groups of genes were compared. These groups were genes that have no seed matches in the 3′UTRs, CDSs, 5′UTRs or Promoters, and genes that have only one seed match in 3′UTRs, CDSs, 5′UTRs and Promoters, respectively. As listed in Table 1, in response to the miRNA overexpression, genes without any seed matches were slightly up-regulated (average log2 protein fold change was 0.041) and only 19% of these genes were down-regulated with a log2 protein fold change less than −0.1, which were considered as true targets. This group of genes was used as the background model of gene expression. Genes with only one seed match in 5′UTRs or Promoters were also slightly up-regulated (average log2 fold change of proteins were 0.044 and 0.043, respectively), and the percentages of true targets in these two groups did not show significant difference compared to the background set by Fisher's exact test. We also found there was no significant difference between seed matches in forward and reverse orientation in the promoter regions (Table S1). Genes containing only one seed match in CDSs had their protein levels slightly up-regulated (average log2 mRNA fold change is 0.029) but had significantly higher percentage of true targets compared to the background set (22.7% of genes had log2 protein fold change less than −0.1, corrected P-value = 3.60E-2 by Fisher's exact test). Genes with one seed match in 3′UTRs had the most down-regulated expression changes after miRNA overexpression and 29.1% of these genes were true targets, significantly higher than the background set (corrected P-value = 3.03E-13 by Fisher's exact test).

**Table 1:** The average log2 protein fold change after miRNA overexpression for each gene group containing seed matches in different gene regions.

Group	Number	Mean	SE	% (<−0.1)	p-value
None	6,405	0.041	0.003	19.2%	
One 3'UTR seed	1,472	−0.014	0.009	29.1%	3.03E-13
One CDS seed	1,566	0.029	0.008	22.7%	3.60E-02
*One 5′UTR seed*	185	0.044	0.026	16.2%	1.00
*One Promoter seed*	3,041	0.043	0.006	18.3%	1.00
One 3'UTR seed+One CDS seed	434	−0.093	0.016	42.2%	2.92E-23
One 3'UTR seed+One 5′UTR seed	70	−0.057	0.031	39.5%	1.74E-03
One 3'UTR seed+One Promoter seed	564	−0.033	0.014	33.3%	6.35E-12
One CDS seed+One 5′UTR seed	179	0.032	0.023	31.6%	3.06E-04
One CDS seed+One Promoter seed	736	0.016	0.010	28.1%	2.40E-06
*One 5'UTR seed+One Promoter seed*	59	0.063	0.031	16.9%	1.00
3'UTRs&CDSs&5'UTRs&Promoters	155	−0.175	0.033	49.6%	<2.2E-16
3'UTRs&CDSs&5'UTRs	274	−0.128	0.020	46.7%	<2.2E-16
3'UTRs&CDSs	3,646	−0.086	0.006	40.9%	<2.2E-16
3'UTRs	8,515	−0.058	0.004	36.2%	<2.2E-16

Number, the total number of genes in each gene group.%(<−0.1), the percentage of genes in the group was down-regulated with a log2 protein fold change less than −0.1 and considered as true targets. P-value, the statistical significance of the proportion of true targets in a target group calculated by the Fisher's exact test and subsequently adjusted for multiple testing using Bonferroni correction. None, genes that have no seed matches of any types. Gene groups in italics indicate the proportion of true target genes in the group is not significantly greater compared to the background model (“None” group).

**Figure 2:**
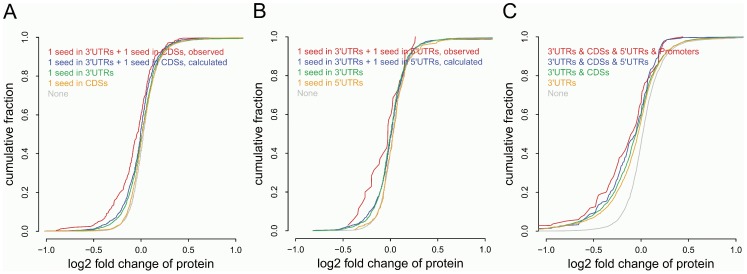
Synergism between target sites located in different gene regions. A) Cumulative distributions of protein log 2 fold changes of genes containing seed matches as indicated. Observed values for genes with one seed in 3′UTRs and one seed in CDSs are in red, simulated values for genes containing two independent seeds in 3′UTRs and CDSs were calculated as described in the Methods section. B) Similar as A) but with 3′UTR and 5′UTR seed combination. C) Cumulative distributions of protein log 2 fold changes of gene groups combining seed matches in multiple regions.

Some previous studies have examined the possible synergistic effects of target sites located in multiple different gene regions [11], [22], [23]. However, it is still unclear how protein output is impacted by the possible interactions between target sites located in different gene regions. To address this issue, we performed the following analyses on log2 protein fold changes and the detailed comparison results are illustrated in Table 1 and Figure 2. For the first analysis, six mutually exclusive groups of genes with dual target sites located in two gene regions were analyzed (Table 1). Compared to the genes containing only one seed match in 3′UTRs, the genes with an additional seed match in a region other than 3′UTR had a higher percentage of true targets (42.2%, 39.5% and 33.3% for genes with an additional seed match in CDSs, 5′UTRs, and Promoter, respectively), indicating seed matches located in regions other than 3′UTRs can enhance miRNA regulatory effects. For some of the dual sites combinations, we found their combinatory effects were significantly more than their expected additive effects. We referred to this more-than-additive effect as “synergistic”. For example, it was observed the repression from genes with one seed match in 3′UTRs and another seed match in CDSs was significantly greater than that expected from the independent contribution of one 3′UTR seed plus one CDS seed (Figure 2A, empirical p-value = 0.010, 1,000 resampling iterations, as described in Methods section). We also observed that, although genes containing only one seed match in 5′UTRs were not significantly down-regulated compared to the background, the genes with an additional seed match in 3′UTRs had a significantly higher percentage of true targets compared to the background model (corrected p-value = 1.74E-03, Fisher's exact test). The repression from these genes with dual sites in 3′ and 5′ UTRs was also significantly greater than those with only one 3′UTR seed match (corrected p-value = 0.036), as well as than that from simulated genes with one 3′UTR plus one 5′UTR site (Figure 2B, empirical p-value = 0.025, 1,000 resampling iterations), indicating seed matches in 3′UTRs have synergistic effects with those in 5′UTRs. This synergistic effect was also observed between seed matches in 3′UTRs and Promoters (empirical p-value = 0.030, 1,000 resampling iterations), as well as between the CDS and promoter sites (empirical p-value = 0.045, 1,000 resampling iterations), leading to a significant contribution to the miRNA regulatory effects (Table 1). For the second analysis on the effects of target sites in multiple gene regions, five groups of genes were compared (Figure 2C). These groups were 1) genes containing seed matches in all of the four gene regions we have studied (3′UTRs, CDSs, 5′UTRs and Promoters), 2) genes containing seed matches in 3′UTRs, CDSs and 5′UTRs, 3) genes containing seed matches both in CDSs and 3′UTRs, 4) genes containing seed matches in 3′UTRs, 5) background genes that have no seed matches in any gene regions (same as above). When genes contain seed matches in all of the four regions we investigated, their protein levels were down-regulated to the lowest level (average log2 fold change of proteins is −0.175) among all the groups we compared. It is observed that gene groups containing target sites located in more gene regions generally demonstrate more significant miRNA regulation effects, as illustrated by their log2 protein fold changes and their percentages of true targets (Table 1). We have noticed that genes with target sites in four different gene regions have at least 4 sites in total, while most genes with target sites limited to just the 3′UTRs have less than 4 sites (Figure S2A). In general, the more target sites the genes have, the more significantly they are down-regulated (Figure S2B). To test that the enhanced miRNA regulatory effect by target sites in multiple gene regions is not simply due to the increased total number of target sites, we stratified genes according to their numbers of miRNA seed matches. It is observed that genes with the same number of seed matches that are located in more gene regions are generally more down-regulated, especially when the number of seed matches they contained is larger than 5, as illustrated by their mean log2 protein fold changes (Figure S2B). All these results above indicate the synergistic effects of miRNA target recognition can be achieved not only by the total number of target sites, but also by combining target sites located in different gene regions.

### Impact of different types of seed matches on protein expression levels

**Figure 3:**
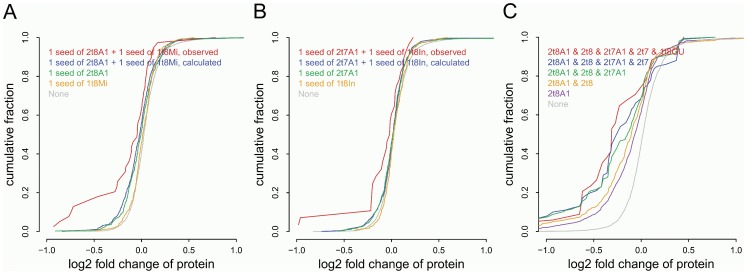
Synergism between different types of seed matches. A) Cumulative distributions of protein log 2 fold changes of genes containing only seed matches as indicated. Observed values for genes with one seed match of 2t8A1 and one seed match of 1t8Mi are in red, simulated values for genes containing two independent seed matches of types 2t8A1 and 1t8Mi were calculated as described in the Methods section. B) Similar as A) but with 2t7A1 and 1t8In seed combination. C) Cumulative distributions of protein log 2 fold changes of genes groups combining multiple types of seed matches.

To investigate miRNA target regulation effects associated with different types of seed matches, we analyzed the log2 protein fold changes for genes with different seed match types in the pSILAC dataset (Table 2, Figure 3) in response to miRNA overexpression. It was found that genes containing one seed match of canonical type 2t8A1 have the highest percentage of true targets, followed by those containing one seed match of type 2t8. Genes with only one seed match of canonical type 2t7A1, 2t7, or non-canonical type were slightly up-regulated and did not show significant miRNA regulation effects compared to the background (Table 2). We also analyzed combinatory effects of dual sites with two different types of seed matches, yielded from totally 28 different combinations (8*7/2). Here we only listed the gene groups with the percentage of true targets significantly higher than that of the background model in Table 2. It was observed that genes with one 1t8Mi seed match were not significantly different from the background (only 19.5% of them are true targets). However, if there is an additional 2t8A1 seed match included in the genes, they would have a significantly higher percentage of true targets compared to the background model (corrected p-value = 3.57E-03, Fisher's exact test). The repression from the genes with dual sites of 2t8A1 and 1t8Mi types was also significantly greater than those with only one 2t8A1 seed match (corrected p-value = 0.042), as well as than that from simulated genes with one 2t8A1 plus one 1t8Mi site (Figure 3A, empirical p-value = 0.038, 1,000 resampling iterations). This more-than-additive effect was also observed for another combination of 2t7A1 and 1t8In seed matches (Figure 3B, empirical p-value = 0.047, 1,000 resampling iterations), suggesting there can be synergistic effects between canonical and non-canonical seed matches.

**Table 2:** The average log2 protein fold change after miRNA overexpression for each gene group containing different types of seed matches.

Group	Number	Mean	SE	% (<−0.1)	p-value
None	6405	0.041	0.003	19.2%	
One 2t8A1	425	−0.049	0.014	35.1%	8.56E-13
One 2t8	929	0.016	0.014	25.9%	1.49E-05
*One 2t7A1*	593	0.002	0.013	22.8%	1.76E-01
*One 2t7*	1,969	0.041	0.007	20.4%	1.00
*One 1t8GU*	1,334	0.050	0.009	18.4%	1.00
*One 1t8Mi*	385	0.006	0.010	19.5%	1.00
*One 1t8In*	169	0.026	0.012	15.4%	1.00
*One 1t8De*	460	0.031	0.016	21.1%	1.00
One 2t8A1+One 2t8	78	−0.175	0.032	43.6%	2.51E-05
One 2t8A1+One 2t7A1	47	−0.078	0.039	40.4%	1.88E-02
One 2t8A1+One 2t7	157	−0.063	0.022	36.3%	1.64E-05
One 2t8A1+One 1t8GU	107	−0.131	0.029	44.9%	5.63E-08
One 2t8A1+One 1t8Mi	79	−0.172	0.061	46.2%	3.18E-04
One 2t8+One 2t7	461	0.031	0.014	28.6%	4.68E-05
One 2t7A1+One 2t7	285	0.005	0.015	27.7%	1.18E-02
One 2t7A1+One 1t8In	58	−0.120	0.060	42.1%	5.68E-04
2t8A1&2t8&2t7A1&2t7&1t8GU	84	−0.264	0.072	67.6%	<2.2E-16
2t8A1&2t8&2t7A1&2t7	100	−0.250	0.052	60.0%	<2.2E-16
2t8A1&2t8&2t7A1	148	−0.250	0.042	53.4%	<2.2E-16
2t8A1&2t8	673	−0.142	0.021	48.4%	<2.2E-16
2t8A1	2,431	−0.099	0.008	41.9%	<2.2E-16

Number, the total number of genes in each gene group.%(<−0.1), the percentage of genes in the group was down-regulated with a log2 protein fold change less than −0.1 and considered as true targets. P-value, the statistical significance of the percentage of true targets in a target group calculated by the Fisher's exact test and subsequently adjusted for multiple testing. Gene groups in italics indicate the percentage of true target genes in the group is not significant compared to the background model (“None” group). For groups with dual sites of different seed match types, only those with the percentage of true targets significantly higher than the background model (corrected p-value<0.05) are listed. The full list of combinations of two seed match types is shown in Table S2.

To further investigate the cooperative effects of multiple types of seed matches, we analyzed and compared genes containing different combinations of multiple seed match types (Figure 3C). In the pSILAC dataset, we didn't find any miRNA targets containing all 8 types of seed matches investigated in this study. The combination of most types of seed matches includes 2t8A1, 2t8, 2t7A1, 2t7 and 1t8GU. Genes with these types of seed matches were down-regulated to the lowest level among all different combinations we have studied (Table 2, Figure 3C). Generally, gene groups containing more types of seed matches demonstrate more significant miRNA regulation effects, as illustrated by their log2 protein fold changes and their percentages of true targets within group. These results imply both canonical and non-canonical seed matches can serve as functional miRNA target sites. miRNAs can enhance their target regulatory effects through binding to sites of different seed match types.

### Site accessibility of miRNA target sites located in different gene regions and containing different types of seed matches

Site accessibility is one of the most important biological factors for miRNAs target recognition [29], [31]. A recent study has demonstrated that site accessibility is selectively varied in the flanking region of miRNA target sites [32]. Here, we used a normalized score Z_ΔΔG_ to measure the extent to which site accessibility of the real gene sequence deviates from random expectation (see Methods section for details). A negative Z_ΔΔG_ means that site accessibility is increased compared to the random expectation, while a positive Z_ΔΔG_ means it is decreased. To further understand the possible underlying mechanism of miRNA target recognition, we calculated the Z_ΔΔG_ along mRNA sequences in sliding windows of 48 nucleotides for each of the experimentally verified miRNA-mRNA pairs from the miRWalk dataset. We started from the miRNA target sites, consisting of 21 nucleotides bound to miRNAs, 17 flank upstream nucleotides and 10 flank downstream nucleotides. Then we moved the sliding window both upwards and downward along the mRNA sequences to calculate the Z_ΔΔG_ for 13 consecutive windows. The miRNA target sites were grouped according to their location in the gene and the types of seed matches they contain. For each window, the mean of Z_ΔΔG_ scores for each group of target sites were calculated and compared.

**Figure 4:**
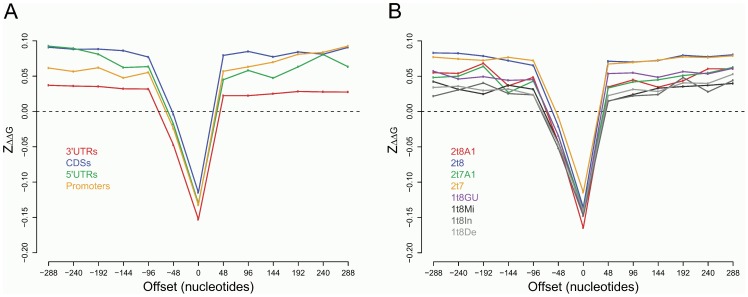
The mean of Z_ΔΔG_ of each sliding window near miRNA target sites. A) Target sites located in different gene regions. B) Target sites of different seed match types.

**Figure 5:**
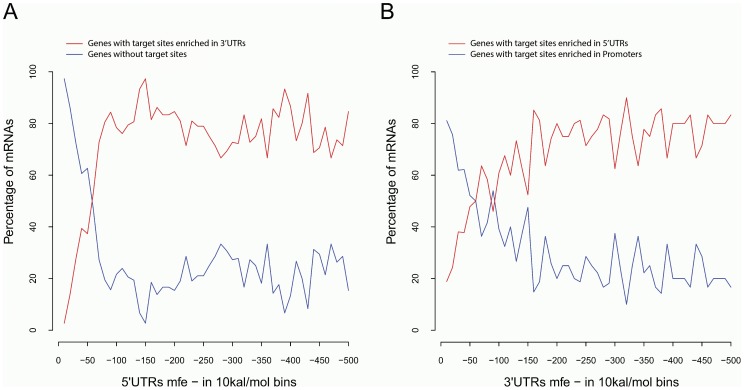
Genes with target sites significantly enriched in different gene regions have different degrees of secondary structure in the 5′UTRs and 3′UTRs. A) Genes with target sites enriched in 3′UTRs have a greater degree of secondary structure in the 5′UTRs than genes without target sites in any region (None). B) Genes with target sites enriched in 5′UTRs have a greater degree of secondary structure in the 3′UTRs than genes with target sites enriched in Promoters.

We compared the scores of Z_ΔΔG_ among target sites located in different gene regions (Figure 4A). It has been observed the increased site accessibility is always present in the central window (miRNA target region), while the decreased site accessibility is generally present in the flank regions of miRNA target sites, regardless of the location of the target sites. However, it is also demonstrated that target sites in 3′UTRs have the most significantly increased site accessibility in the target region and the least significantly decreased site accessibility along the nearby flank regions, while target sites in CDSs have the least significantly increased site accessibility in the target region and relatively high decreased site accessibility along the nearby flank regions (Figure 5A). Target sites in 5′UTRs and Promoters indicate site accessibility changes at intermediate levels between those in CDSs and 3′UTRs. These results together suggest the importance of site accessibility in affecting miRNA regulation among different gene regions. Site accessibility is a dominant factor when target sites are located in 3′UTRs. For target sites in CDSs, there could be other factors affecting miRNA action, such as the local translation efficiency, as suggested by [32], [33].

We also compared the scores of Z_ΔΔG_ among target sites with different types of seed matches (Figure 4B). In the target region, it is observed that seed match 2t8A1 has the most significantly increased site accessibility, while other canonical seed matches (2t8, 2t7A1 and 2t7) have least increased site accessibility and the non-canonical seed matches have site accessibility changes at intermediate levels. Along the nearby flank regions, we have demonstrated that the non-canonical seed matches, except 1t8GU, have less significantly decreased site accessibility than the canonical seed matches. This result suggests the site accessibility is varied among different types of seed matches. Target sites containing non-canonical seed matches or the canonical seed match type 2t8A1 might lead to loose mRNA structures in the target regions, compared to other types of seed matches.

### Secondary structure of mRNAs with target sites in different regions

A recent study has shown that mRNAs with miRNA target sites in their 3′UTRs have a greater degree of secondary structure in the 5′UTRs than do mRNAs without miRNA target sites [34]. In this study, we further compared the distal secondary structure features of genes with their target sites located in different regions. Because the number of experimentally validated miRNA targets from miRWalk is insufficient for this analysis, we extracted the targets information from the TargetS database, which was built on the computationally predicted results on the complete sequences (promoter, 5' UTR, CDS and 3' UTR) of all known human genes [29]. It has been observed that one mRNA can be regulated by multiple miRNAs, with the corresponding target sites located in different gene regions. In this analysis, we only considered those genes with target sites significantly enriched in only one of the four gene regions we investigated (See Methods section for details). The genes predicted to have target sites either significantly enriched in multiple gene regions or not significantly enriched in any of the regions were excluded from our following analysis. Therefore, we have five mutually exclusive classes of genes: 1) genes not targeted by any miRNAs (2682 genes), 2) genes having target sites significantly enriched in 3′UTRs only (1258 genes), 3) genes having target sites significantly enriched in CDSs only (603 genes), 4) genes having target sites significantly enriched in 5′UTRs only (854 genes), and 5) genes having target sites significantly enriched in Promoters only (842 genes). The target gene sequences in each class were analyzed for their distal secondary structure and grouped into 10 kcal/mol bins.

From Figure 5A, we can see that genes with predicted target sites enriched in 3′UTRs have a greater degree of secondary structure in the 5′UTRs than do genes without target sites in any regions, which is consistent with [34]. It is also demonstrated that these genes have a greater degree of secondary structure in the 5′UTRs than those with predicted target sites enriched in CDSs or Promoters only (Figure S3). We next compared the secondary structure in the 3′UTRs for different groups of genes. Genes with predicted target sites enriched in 5′UTRs have a greater degree of secondary structure in 3′UTRs than those with predicted target sites enriched in Promoters or CDSs (Figure 5B, Figure S4A). Interestingly, there is no significant difference for the degree of secondary structure in 3′UTRs (Figure S4B) or 5′UTRs (Figure S4C) between genes with predicted target sites enriched in CDSs and those with predicted target sites enriched in Promoters. These results together suggest the contribution of secondary structure in 3′UTRs or 5′UTRs on miRNA targeting is different among gene groups with different target site locations.

## Discussion

While it is clear that miRNAs play key roles in post-transcriptionally regulating gene expression, the mechanisms by which miRNAs bind and recognize their targets are still poorly understood. Many of the current studies have focused primarily on the seed matches between miRNAs and targets, as well as the mRNA secondary structures in which the target sites are located. However, current work in the field is often limited to studying miRNA target sites containing canonical types of seed matches and neglects the non-canonical ones. In addition, there are only a few studies that investigated the impact of miRNA target sites when they are located in CDSs of genes [11], [22], [23], while most studies only focused on the effects of target sites located in the 3′UTRs of genes. In this study, to comprehensively investigate how miRNA regulatory effects are impacted by a wide spectrum of seed matches, we introduced four additional non-canonical types of seed matches beyond the four canonical ones, and we extended the regions of target sites to include 3′UTRs, CDSs, 5′UTRs and Promoters. The miRNA targets were grouped according to the types of seed matches they contain, as well as the location of these seed matches. We analyzed the pSILAC dataset to investigate the impact of seed matches on miRNA regulatory effects. It is noticed there are only five miRNAs included in the pSILAC experiment and there is much variability between these miRNAs in terms of their target sites location and the types of seed matches they contain, as shown in Tables S3 and S4. For example, compared to all the miRNAs included in miRWalk, miR16 has seed matches significantly enriched in the CDSs of target genes, while let7b and miR1 have seed matches more significantly enriched in the promoter regions of their target genes. When the information of miRNA-target pairs for all the five miRNAs was pooled together, the distribution of seed matches in the four gene regions was similar to what was found for all the miRNAs in miRWalk (Table S3). Therefore, we believe the five miRNAs can well represent other human miRNAs when being analyzed for the impact of target site locations on protein expression levels. However, when we compared the seed matches types of the five miRNAs in pSILAC with those in miRWalk, we found these five miRNAs have significantly more canonical seed matches but less non-canonical seed matches, especially for types 1t8Mi, 1t8In and 1t8De, than the whole set of human miRNAs in miRWalk (Table S4). Therefore, our analysis on the three non-canonical seed match types might be limited due to the limited number of miRNAs in the pSILAC experiment. Nevertheless, our results demonstrate that seed matches of different types or their locations within different gene regions contribute to the strength of miRNA-mediated responses differently. A possible explanation of the difference is the structural features exhibited by the target sites containing these seed matches, such as the site accessibility of the flanking region of the target sites and the secondary structure of the gene regions distant from the target site.

We performed a genome-wide analysis to investigate the possible synergistic effects of target sites located in different gene regions, or seed matches of different types on miRNA regulation, quantified by the changes of protein expression levels in response to over-expressed miRNAs. It has been clearly demonstrated that target sites located in different gene regions act synergistically to enhance the miRNA regulation efficiency. It was also observed that genes containing both canonical seed matches and non-canonical ones were significantly more regulated than those containing canonical seed matches only, indicating both canonical and non-canonical seed matches can be functional. This synergistic rule can be applied when identifying miRNA targets to increase the accuracy of miRNA target identification methods. For example, taking into account the fact there are synergistic effects among target sites located in different gene regions, we can modify the target selection criteria of the TargetS method [29] we developed previously to increase the sensitivity and precision level of our method. If we added a requirement that the targets must contain seed matches in 3′UTRs and in at least one of other regions (CDSs, 5′UTRs, or Promoters), our method can achieve a precision level of 61.3% (623/1017) using the pSILAC dataset as the benchmark. The precision level of our modified TargetS method is much higher than that of the TargetScan (56.3%) [35] and MicroT_CDS (57.2%) [22], as shown in Table 3. The precision level of our method is comparable with that of PicTar (64.5%, 258/400) [36], but the total number of true targets we identified is 2.5 times more than that of PicTar (Table 3).

**Table 3:** A performance comparison of different computational miRNA target identification methods.

Methods	Number of True Positives	Number of False Positives	Number of False Negatives	Sensitivity	Precision
TargetS	623	394	5927	0.10	0.61
TargetScanS	545	422	6005	0.08	0.56
MicroT_CDS	536	401	6014	0.08	0.57
PicTar	258	142	6292	0.04	0.64

The methods were evaluated by the independent benchmark dataset obtained by the pSILAC. Sensitivity, the proportion of true targets that are identified. Precision, the proportion of identified targets that are true targets.

To investigate the biological factors affecting miRNA action on targets with different types and locations of target sites, we have performed a genome scale analysis for studying site accessibility near miRNA target regions. We have found that site accessibility is selectively varied in the flank region of miRNA target sites and tends to be higher in miRNA target regions than expected by chance. More importantly, we observed different levels of site accessibility among target sites located in different gene regions or of different seed match types. A possible explanation of the difference is that human miRNAs regulate targets under different mechanisms, depending on the location of the target sites and on the type of seed matches the target sites contain. For example, when target sites are located in 3′UTRs, where the site accessibility is most significantly increased in the target region, miRNAs might affect mRNA stability that eventually leads to mRNA degradation. For genes containing target sites in CDSs, miRNA regulate them preferably by inhibiting mRNA translation. Similarly, the target sites containing non-canonical seed matches or canonical seed match type 2t8A1 might be more potent in triggering mRNA degradation as their site accessibility are more increased than other canonical types of seed matches.

We further compared the secondary structure features of genes with their target sites located in different gene regions. Information on miRNA targets were extracted from the TargetS database and genes with predicted target sites significantly enriched in each of the four gene regions were compared. From the analysis, we can see that genes with predicted target sites enriched in 3′UTRs have a greater degree of secondary structure in 5′UTRs than genes with predicted target sites enriched in CDSs, 5′UTRs, or Promoters. It was also shown that genes with predicted target sites enriched in 5′UTRs have a greater degree of secondary structure in 3′UTRs than those with predicted target sites enriched in Promoters or CDSs. These results suggest that miRNA targeting on 3′UTRs is dependent on the secondary structure of 5′UTRs. Similarly, miRNA-mediated gene regulation on the genes with target sites enriched in 5′UTRs may rely on the secondary structure of 3′UTRs. It implies that 3′UTRs and 5′UTRs have structure relationships and can function cooperatively, and they may prefer being synergistic among all the gene regions to enhance the miRNA targeting function.

In conclusion, miRNA target binding and recognition can be achieved under different mechanisms, depending on the locations of target sites and the types of seed matches the target sites contain. This new discovery shed lights on the problem of current miRNA target prediction algorithms that might lack of full picture of the regulatory mechanisms. Our future work will focus on improving miRNA prediction accuracy by integrating miRNA sequence feature and mRNA expression profiling using the framework we proposed [37]. In addition, the results from this study may help us better understand the functional insights of miRNA actions. Future work will investigate different families of miRNAs that show distinct targeting site location/type preferences and determine their roles involved in regulating different biological processes.

## Materials and Methods

### Data

miRWalk: This dataset hosts experimentally verified miRNA-mRNA interactions [30]. It includes 60,269 verified pairs of human miRNA-gene interactions that consist of 655 unique miRNAs and 3,028 unique genes.

pSILAC: A set of miRNA target genes identified by pSILAC (pulsed stable isotope labeling with amino acids in cell culture) method [7]. It measured changes in synthesis of several thousand proteins in response to miRNA transfection for five miRNAs (hsa-miR-1, hsa-miR-16, hsa-miR-155, hsa-miR-30a and hsa-let-7b) and the endogenous miRNA knock-down for has-let-7b. In this study, we applied our analysis on the dataset obtained from the miRNA overexpression experiments for all the five miRNAs. This dataset has been widely used as a benchmark for evaluating computational miRNA target prediction programs.

miRBase: The mature miRNAs sequences are downloaded from miRBase database [6]. There are more than 30,000 reported miRNAs entries, including 2,557 entries for human in the latest version (Release 20, 2013).

Sequences: The sequences of the Promoters, 5′UTRs, CDSs and 3′UTRs for each gene in human have been downloaded from the UCSC Genomes database [38] using the UCSC Table Brower, version GRCh37/hg19. When there are multiple sequences available for a single gene (e.g. multiple UCSC IDs corresponds to a single gene name), the longest sequence was chosen for further analysis.

### Signal-to-noise ratio calculation

The signal-to-noise ratio compares the signal of real miRNA target regions in the mRNA sequences to that from background noise. The miRNAs sequences were downloaded from miRBase and the mRNA sequences were downloaded from the UCSC Genomes database. We calculated the signal-to-noise ratio for each type of seed matches located in different regions. Based on the verified miRNA-mRNA pairs in the miRWalk dataset, we counted the number of seed matches of each type in different gene regions, and then randomly permuted the mRNAs sequence 50 times and computed the average numbers of each type of these seed matches occurring in the 50 permuted sequences.

### Simulating miRNA regulatory effects for dual sites

We simulated the cumulative distribution of changes in protein levels for genes containing two miRNA binding sites, similar to that in [16]. For example, the simulated dual-site distribution (one seed in 3′UTRs plus one seed in CDSs, blue line in Figure 2A) was derived by randomly selecting one gene with only one 3′UTR seed match and another gene with only one CDS seed match and summing their log2 protein expression changes. This procedure was repeated 1,000 times for simulating the dual-site distribution. The percentage of log2 protein fold changes less than −0.1 in these simulated gene groups was calculated. For comparison to the simulated distributions, the observed dual-site distribution was modified by randomly selecting one gene containing both a 3′UTR seed and a CDS seed and one gene that have no seed match and summing their log2 expression changes (red line, Figure 2A). The empirical p-value of the dual-site synergistic effect was calculated as the fraction of the 1,000 simulations having a larger percentage of log2 protein fold changes less than −0.1 than that based on the observed data.

### Site accessibility and Z-score calculation

Site accessibility has been used to measure the degree of difficulty in opening the mRNA sequences for miRNA targeting and binding. We used the accessibility energy ΔΔG introduced by [31] to measure the site accessibility for miRNA target sites of different seed match types and located in different gene regions. The metrics incorporates both the binding energy of forming the miRNA-target duplex (ΔG_duplex_), and the energy cost of making the target site accessible (ΔG_open_), including the cost of unpairing additional bases flanking downstream and upstream of the target site. The accessibility energy ΔΔG is the difference between the binding energy ΔG_duplex_, calculated by RNAhybrid [39], and the free energy to unpair the target site nucleotides ΔG_open_, calculated by RNAfold [40]. Each target site consists of 21 nucleotides bound to miRNAs, 17 flank upstream nucleotides and 10 flank downstream nucleotides. In this study, we used the Z-score of ΔΔG to measure the extent to which the site accessibility deviates from random expectation, similar to that in [32]. The Z-score of ΔΔG is defined in equation (1). 
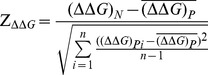
(1)


Here, 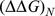
is site accessibility for the naturally occurring target region under consideration. 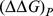 is the mean of 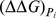 which is for the target region in the *i^th^* permuted sequence. *n* is the total number of permuted sequences, and is equal to 1,000 in this study.

### Secondary structure analysis

The mRNA sequences were analyzed by using RNAfold [40] for potential secondary structure and grouped into 10 kcal/mol-wide minimum free-energy (mfe) bins ranging from 0 to −500 kcal/mol. The mRNAs within each bin were analyzed separately depending on the gene region where miRNA seed matches were significantly enriched in. We first determined whether the seed matches tend to be located in a specific gene region more than expected by chance for each miRNA-target pair from the TargetS method we developed previously [29]. For the miRNA-target pair *i*, we counted the number of seed matches in gene region *j* as *S_ij_* for each of the four gene regions. The fold enrichment of *S_ij_* is defined as 
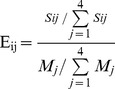
(2)


Here, *M_j_* represents the number of seed matches located in region *j* for all the miRNA-target pairs. We used Fisher's test to assess whether the obtained fold enrichment is significant different from 1. The P-value was corrected for multiple testing using Bonferroni correction. The gene would be considered to have seed matches significantly enriched in region *j* compared to the overall distribution of seed matches among four gene regions for all the target genes if the corrected P-value is less than 0.05.

## Supporting Information

Figure S1 Eight different seed matches types. A–D) Canonical seed matches types. E–H) Non-canonical seed matches types.
(TIF)

Figure S2 The number of seed matches and the average log2 protein fold changes for each gene group combining target sites located in different regions. A) Distribution of genes in each group according to the total number of seed matches they contain. B) The average log2 protein fold changes for genes with corresponding numbers of seed matches.
(TIF)

Figure S3 Secondary structure in 5′UTRs. Genes with target sites significantly enriched in 3′UTRs have a greater degree of secondary structure in the 5′UTRs than genes with target sites enriched in CDSs or Promoters.
(TIF)

Figure S4 Secondary structure in 3′UTRs and 5′UTRs. A) Genes with target sites significantly enriched in 5′UTRs have a greater degree of secondary structure in 3′UTRs than genes with target sites enriched in CDSs. B–C) There is no significant difference for the degree of secondary structure in 3′UTRs or 5′UTRs, between genes having target sites enriched in CDSs and those having target sites enriched in Promoters.
(TIF)

Table S1 The average log2 protein fold changes for gene groups containing seed matches in promoter regions in forward and reverse orientation. Number, the total number of genes in each gene group.%(<−0.1), the percentage of genes in the group was down-regulated with a log2 protein fold change less than −0.1. None, genes that have no seed match in any gene regions.
(DOCX)

Table S2 The average log2 protein fold change for each gene group containing dual sites of different seed match types. Number, the total number of genes in each gene group.%(<−0.1), the percentage of genes in the group was down-regulated with a log2 protein fold change less than −0.1 and considered as true targets. P-value, the statistical significance of the percentage of true targets in a target group calculated by the Fisher's exact test and subsequently adjusted for multiple testing using Bonferroni correction. None, genes that have no seed matches of any types. Gene groups in bold indicate the proportion of true target genes in the group is significantly greater compared to the background model (“None” group).
(DOCX)

Table S3 Distribution of seed matches among different gene regions for 5 miRNAs (let7b, miR16, miR1, miR155 and miR30a). Numbers in the parenthesis represent the percentage of seed matches in an indicated gene region for each miRNA. Pooled, percentage of seed matches in a gene region for all five miRNAs. miRWalk, percentage of seed matches in a gene region for all miRNAs in miRWalk.
(DOCX)

Table S4 Numbers of different types of seed matches for 5 miRNAs (let7b, miR16, miR1, miR155 and miR30a). Numbers in the parenthesis represent the percentage of seed matches of an indicated type for each miRNA. Pooled, percentage of different types of seed matches for all five miRNAs. miRWalk, percentage of different types of seed matches for all miRNAs in miRWalk.
(DOCX)
