# Supplementary figures and images for: The Characterization of microRNA-Mediated Gene Regulation as Impacted by Both Target Site Location and Seed Match Type
Source: PLoS One. 2014 Sep 19;9(9):e108260. doi: 10.1371/journal.pone.0108260 (PMC4169588; doi:10.1371/journal.pone.0108260)

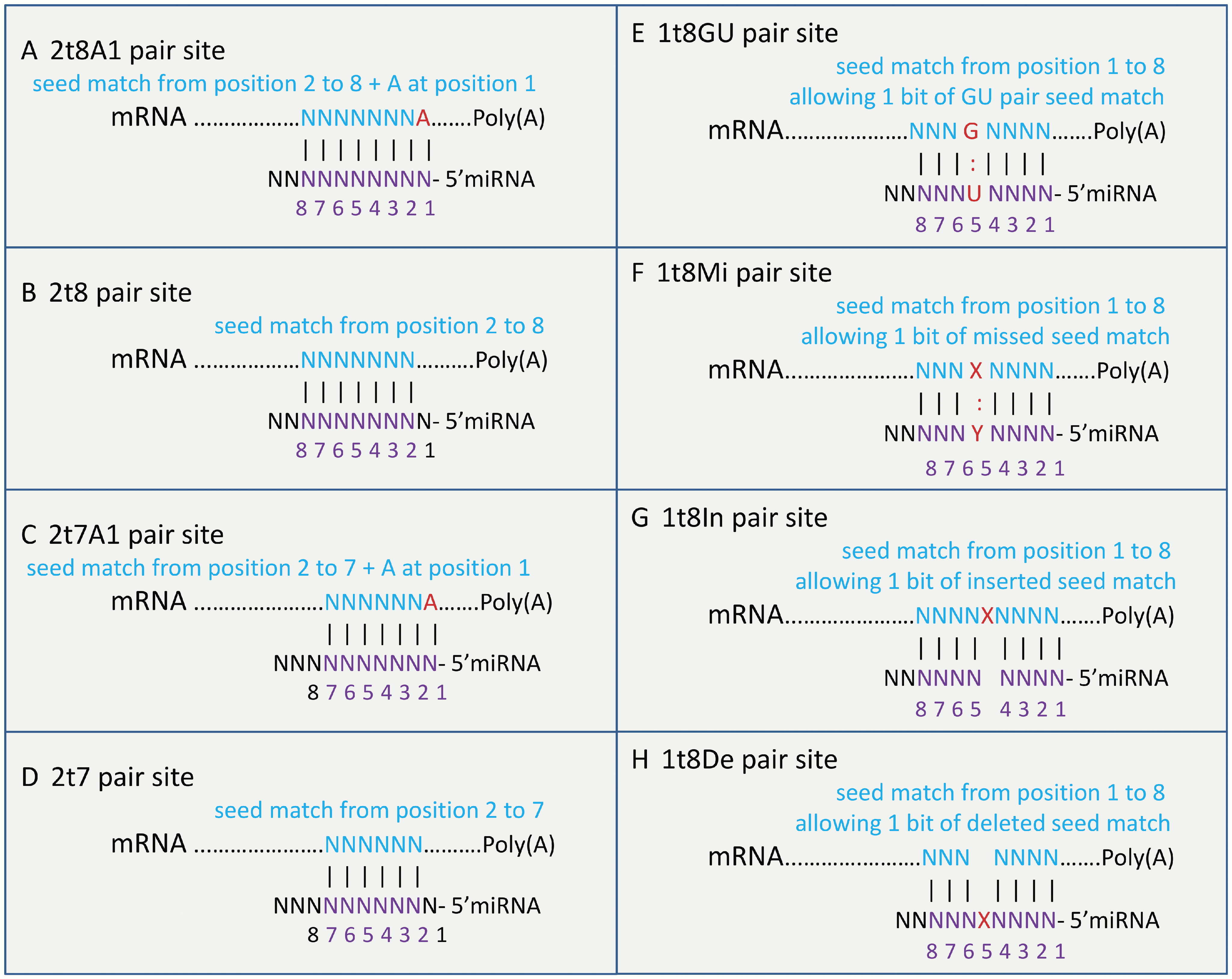

Supplement: Figure S1 — Eight different seed matches types. A–D) Canonical seed matches types. E–H) Non-canonical seed matches types. (TIF) [file pone.0108260.s001.tif]

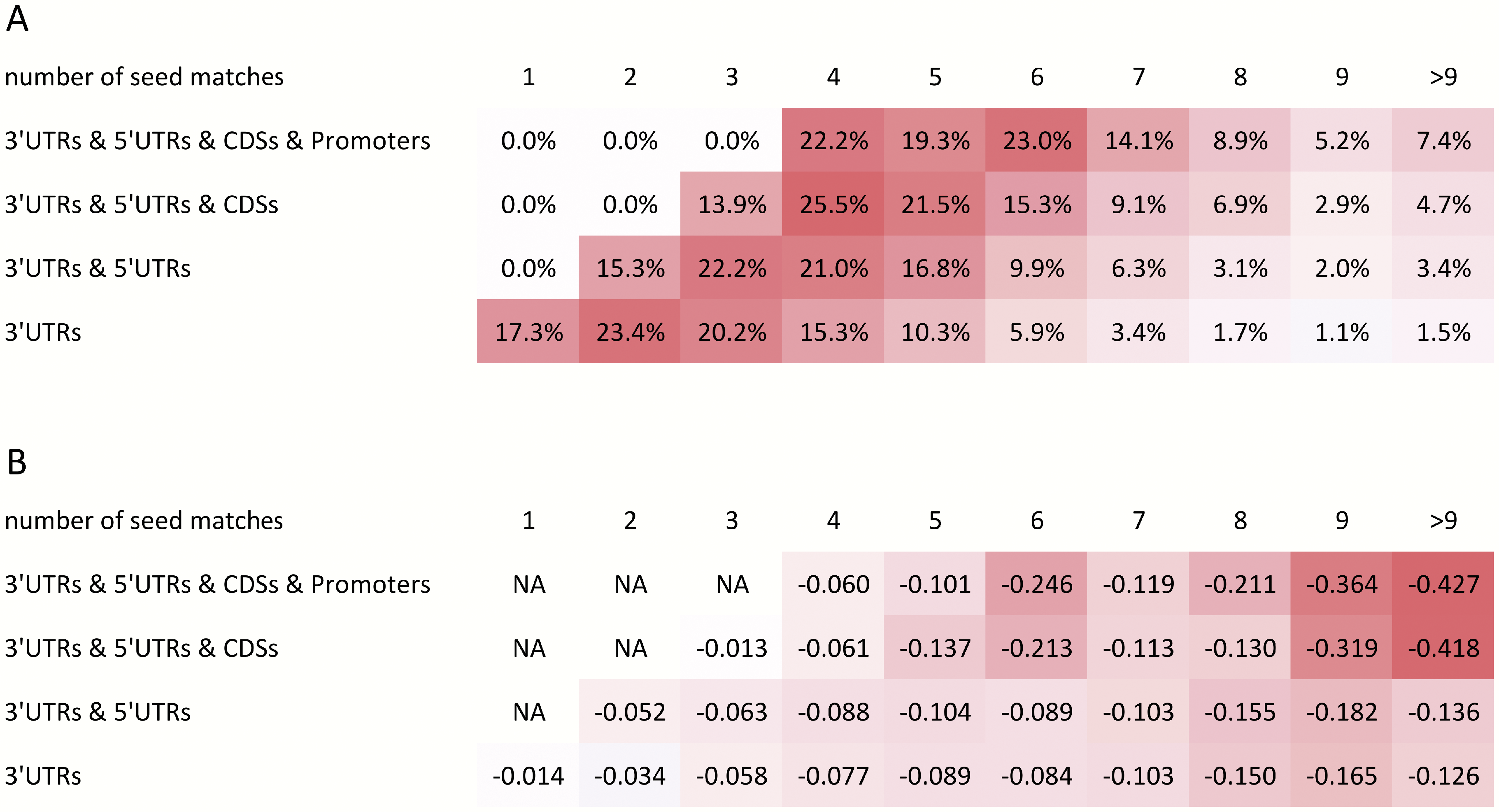

Supplement: Figure S2 — The number of seed matches and the average log2 protein fold changes for each gene group combining target sites located in different regions. A) Distribution of genes in each group according to the total number of seed matches they contain. B) The average log2 protein fold changes for genes with corresponding numbers of seed matches. (TIF) [file pone.0108260.s002.tif]

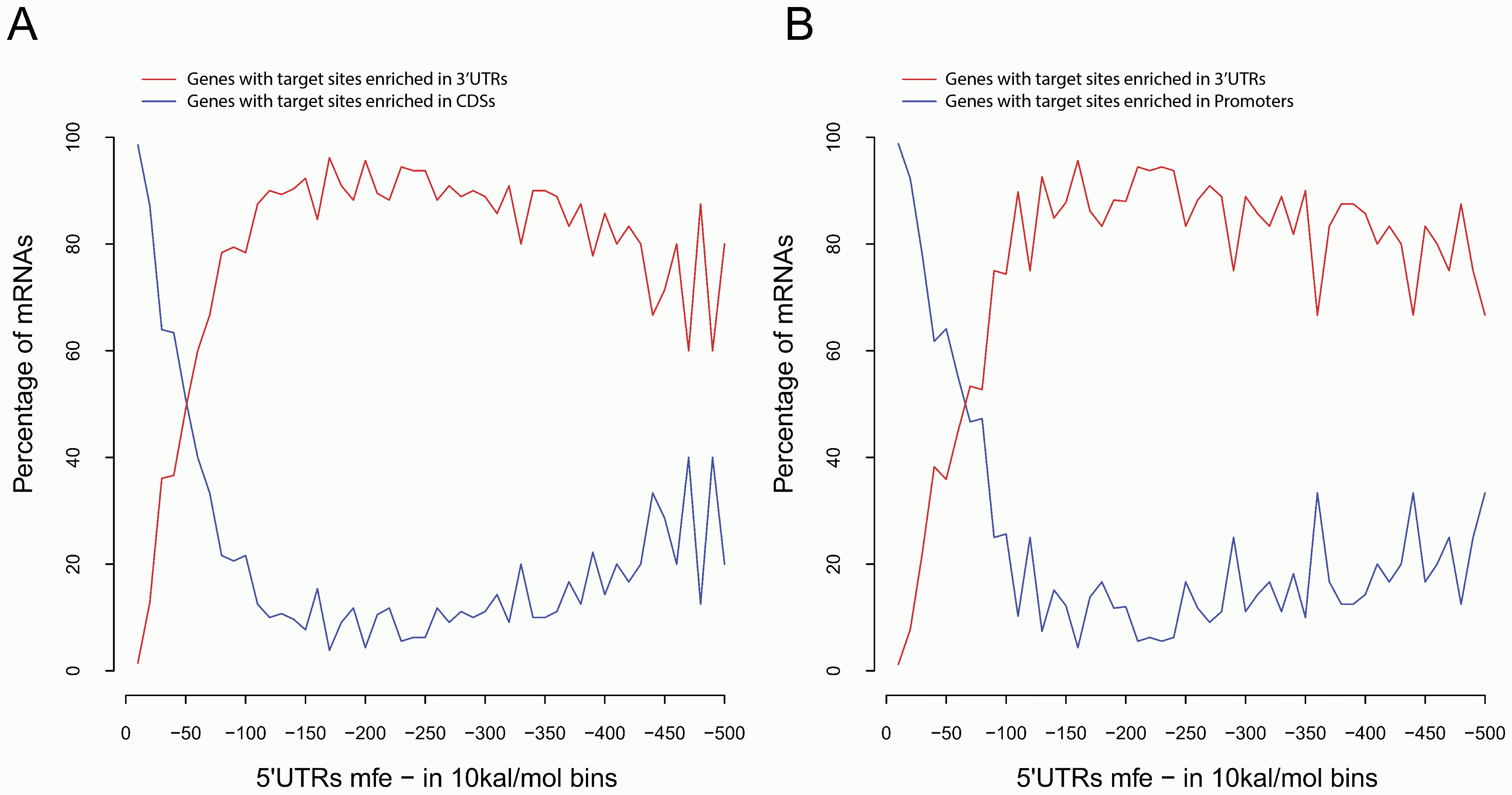

Supplement: Figure S3 — Secondary structure in 5′UTRs. Genes with target sites significantly enriched in 3′UTRs have a greater degree of secondary structure in the 5′UTRs than genes with target sites enriched in CDSs or Promoters. (TIF) [file pone.0108260.s003.tif]

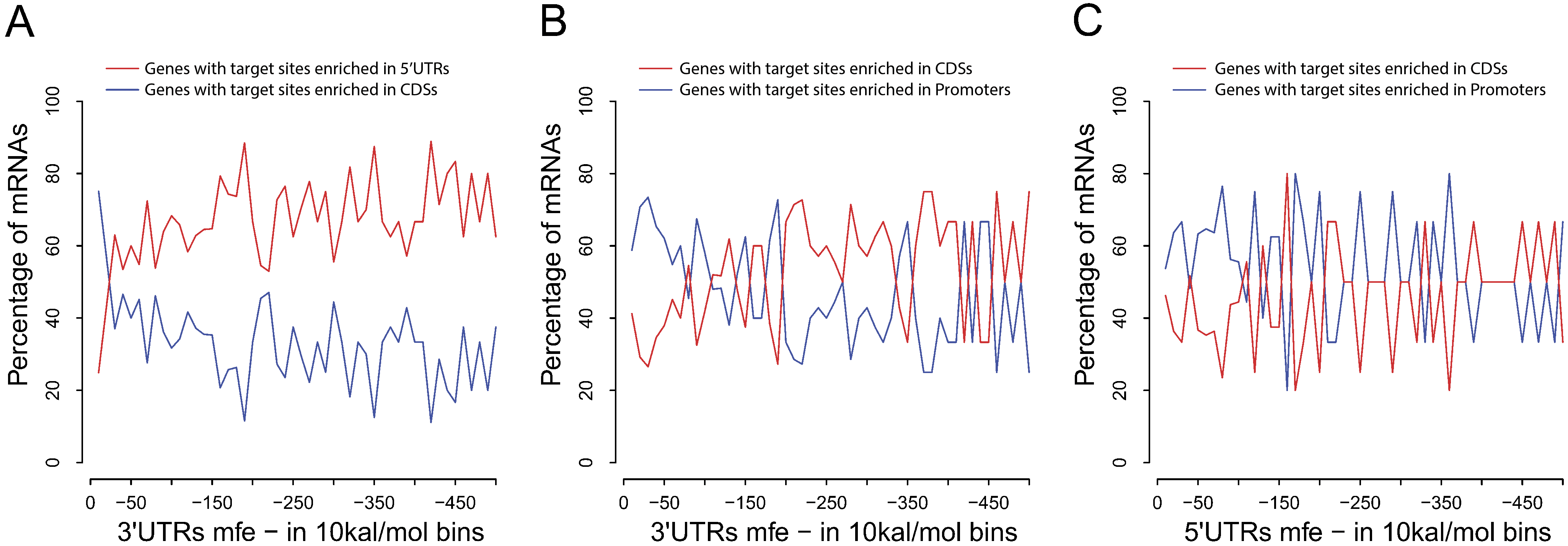

Supplement: Figure S4 — Secondary structure in 3′UTRs and 5′UTRs. A) Genes with target sites significantly enriched in 5′UTRs have a greater degree of secondary structure in 3′UTRs than genes with target sites enriched in CDSs. B–C) There is no significant difference for the degree of secondary structure in 3′UTRs or 5′UTRs, between genes having target sites enriched in CDSs and those having target sites enriched in Promoters. (TIF) [file pone.0108260.s004.tif]
