# Supplementary material for: The Characterization of microRNA-Mediated Gene Regulation as Impacted by Both Target Site Location and Seed Match Type
Source: PLoS One. 2014 Sep 19;9(9):e108260. doi: 10.1371/journal.pone.0108260 (PMC4169588; doi:10.1371/journal.pone.0108260)
Supplement: Table S1 — The average log2 protein fold changes for gene groups containing seed matches in promoter regions in forward and reverse orientation. Number, the total number of genes in each gene group.%(<−0.1), the percentage of genes in the group was down-regulated with a log2 protein fold change less than −0.1. None, genes that have no seed match in any gene regions. (DOCX) [file pone.0108260.s005.docx]

**Table S1. The average log2 protein fold changes for gene groups containing seed matches in promoter regions in forward and reverse orientation.** Number, the total number of genes in each gene group. %(<-0.1), the percentage of genes in the group was down-regulated with a log2 protein fold change less than -0.1. None, genes that have no seed match in any gene regions.

| Group | Number | Mean | SE | % (< -0.1) |
| --- | --- | --- | --- | --- |
| None | 6,405 | 0.041 | 0.003 | 19.2% |
| Forward | 2,290 | 0.039 | 0.005 | 18.9% |
| Reverse | 2,334 | 0.051 | 0.006 | 19.3% |
