# Supplementary material for: The Characterization of microRNA-Mediated Gene Regulation as Impacted by Both Target Site Location and Seed Match Type
Source: PLoS One. 2014 Sep 19;9(9):e108260. doi: 10.1371/journal.pone.0108260 (PMC4169588; doi:10.1371/journal.pone.0108260)
Supplement: Table S2 — The average log2 protein fold change for each gene group containing dual sites of different seed match types. Number, the total number of genes in each gene group.%(<−0.1), the percentage of genes in the group was down-regulated with a log2 protein fold change less than −0.1 and considered as true targets. P-value, the statistical significance of the percentage of true targets in a target group calculated by the Fisher's exact test and subsequently adjusted for multiple testing using Bonferroni correction. None, genes that have no seed matches of any types. Gene groups in bold indicate the proportion of true target genes in the group is significantly greater compared to the background model (“None” group). (DOCX) [file pone.0108260.s006.docx]

**Table S2. The average log2 protein fold change for each gene group containing dual sites of different seed match types.** Number, the total number of genes in each gene group. %(< -0.1), the percentage of genes in the group was down-regulated with a log2 protein fold change less than -0.1 and considered as true targets. P-value, the statistical significance of the percentage of true targets in a target group calculated by the Fisher’s exact test and subsequently adjusted for multiple testing using Bonferroni correction. None, genes that have no seed matches of any types. Gene groups in bold indicate the proportion of true target genes in the group is significantly greater compared to the background model (“None” group).

| Group | Number | Mean | SE | %(<-0.1) | p-value |
| --- | --- | --- | --- | --- | --- |
| None | 6495 | 0.041 | 0.003 | 19.2% |  |
| **1 2t8A1 + 1 2t8** | **78** | **-0.175** | **0.032** | **43.6%** | **2.51E-05** |
| **1 2t8A1 + 1 2t7A1** | **47** | **-0.078** | **0.039** | **40.4%** | **1.88E-02** |
| **1 2t8A1 + 1 2t7** | **157** | **-0.063** | **0.022** | **36.3%** | **1.64E-05** |
| **1 2t8A1 + 1 1t8GU** | **107** | **-0.131** | **0.029** | **44.9%** | **5.63E-08** |
| **1 2t8A1 + 1 1t8Mi** | **79** | **-0.172** | **0.061** | **46.2%** | **3.18E-06** |
| 1 2t8A1 + 1 1t8In | 11 | -0.160 | 0.106 | 54.5% | 2.69E-01 |
| 1 2t8A1 + 1 1t8De | 55 | 0.033 | 0.029 | 25.5% | 1.00 |
| 1 2t8 + 1 2t7A1 | 134 | -0.005 | 0.029 | 29.1% | 1.15E-01 |
| **1 2t8 + 1 2t7** | **461** | **0.031** | **0.014** | **28.6%** | **4.68E-05** |
| 1 2t8 + 1 1t8GU | 245 | 0.025 | 0.027 | 26.9% | 6.74E-02 |
| 1 2t8 + 1 1t8Mi | 82 | 0.132 | 0.120 | 22.0% | 1.00 |
| 1 2t8 + 1 1t8In | 33 | 0.096 | 0.031 | 24.2% | 1.00 |
| 1 2t8 + 1 1t8De | 95 | -0.013 | 0.026 | 31.6% | 8.33E-02 |
| **1 2t7A1 + 1 2t7** | **285** | **0.005** | **0.015** | **27.7%** | **1.18E-02** |
| 1 2t7A1 + 1 1t8GU | 174 | -0.018 | 0.030 | 27.6% | 1.44E-01 |
| 1 2t7A1 + 1 1t8Mi | 76 | -0.043 | 0.026 | 30.3% | 4.05E-01 |
| **1 2t7A1 + 1 1t8In** | **58** | **-0.120** | **0.060** | **42.1%** | **5.68E-04** |
| 1 2t7A1 + 1 1t8De | 69 | -0.057 | 0.024 | 21.7% | 1.00 |
| 1 2t7 + 1 1t8GU | 541 | 0.025 | 0.012 | 24.4% | 7.06E-02 |
| 1 2t7 + 1 1t8Mi | 229 | 0.021 | 0.017 | 23.6% | 1.00 |
| 1 2t7 + 1 1t8In | 71 | 0.108 | 0.063 | 22.5% | 1.00 |
| 1 2t7 + 1 1t8De | 200 | 0.079 | 0.014 | 17.5% | 1.00 |
| 1 1t8GU + 1 1t8Mi | 159 | 0.020 | 0.015 | 27.7% | 1.87E-01 |
| 1 1t8GU + 1 1t8In | 100 | -0.021 | 0.029 | 26.0% | 1.00 |
| 1 1t8GU + 1 1t8De | 183 | 0.017 | 0.019 | 21.9% | 1.00 |
| 1 1t8Mi + 1 1t8In | 27 | 0.024 | 0.023 | 11.1% | 1.00 |
| 1 1t8Mi + 1 1t8De | 43 | 0.124 | 0.031 | 7.0% | 1.00 |
| 1 1t8In + 1 1t8De | 36 | 0.061 | 0.044 | 16.7% | 1.00 |
