# Supplementary material for: The Characterization of microRNA-Mediated Gene Regulation as Impacted by Both Target Site Location and Seed Match Type
Source: PLoS One. 2014 Sep 19;9(9):e108260. doi: 10.1371/journal.pone.0108260 (PMC4169588; doi:10.1371/journal.pone.0108260)
Supplement: Table S3 — Distribution of seed matches among different gene regions for 5 miRNAs (let7b, miR16, miR1, miR155 and miR30a). Numbers in the parenthesis represent the percentage of seed matches in an indicated gene region for each miRNA. Pooled, percentage of seed matches in a gene region for all five miRNAs. miRWalk, percentage of seed matches in a gene region for all miRNAs in miRWalk. (DOCX) [file pone.0108260.s007.docx]

**Table S3. Distribution of seed matches among different gene regions for 5 miRNAs (let7b, miR16, miR1, miR155 and miR30a).** Numbers in the parenthesis represent the percentage of seed matches in an indicated gene region for each miRNA. Pooled, percentage of seed matches in a gene region for all five miRNAs. miRWalk, percentage of seed matches in a gene region for all miRNAs in miRWalk.

| Region | let7b | miR16 | miR1 | miR155 | miR30a | Pooled | miRWalk |
| --- | --- | --- | --- | --- | --- | --- | --- |
| 3’UTRs | 581(28%) | 734(27%) | 661(32%) | 636(38%) | 471(40%) | 33% | 34% |
| CDSs | 594(28%) | 997(37%) | 619(30%) | 474(29%) | 282(24%) | 30% | 30% |
| 5’UTRs | 73(4%) | 223(8%) | 68(3%) | 33(2%) | 29(2%) | 4% | 5% |
| Promoters | 864(40%) | 744(28%) | 742(35%) | 521(31%) | 405(34%) | 33% | 31% |
