# Supplementary material for: The Characterization of microRNA-Mediated Gene Regulation as Impacted by Both Target Site Location and Seed Match Type
Source: PLoS One. 2014 Sep 19;9(9):e108260. doi: 10.1371/journal.pone.0108260 (PMC4169588; doi:10.1371/journal.pone.0108260)
Supplement: Table S4 — Numbers of different types of seed matches for 5 miRNAs (let7b, miR16, miR1, miR155 and miR30a). Numbers in the parenthesis represent the percentage of seed matches of an indicated type for each miRNA. Pooled, percentage of different types of seed matches for all five miRNAs. miRWalk, percentage of different types of seed matches for all miRNAs in miRWalk. (DOCX) [file pone.0108260.s008.docx]

**Table S4. Numbers of different types of seed matches for 5 miRNAs (let7b, miR16, miR1, miR155 and miR30a).** Numbers in the parenthesis represent the percentage of seed matches of an indicated type for each miRNA. Pooled, percentage of different types of seed matches for all five miRNAs. miRWalk, percentage of different types of seed matches for all miRNAs in miRWalk.

| Type | let7b | miR16 | miR1 | miR155 | miR30a | Pooled | miRWalk |
| --- | --- | --- | --- | --- | --- | --- | --- |
| 2t8A1 | 208 (8%) | 205(8%) | 196(10%) | 184(6%) | 225(14%) | 8% | 3% |
| 2t8 | 359(13%) | 364(14%) | 370(19%) | 819(27%) | 223(14%) | 18% | 7% |
| 2t7A1 | 166(6%) | 290(11%) | 241(12%) | 320(10%) | 215(13%) | 10% | 5% |
| 2t7 | 472(17%) | 629(23%) | 559(28%) | 1141(37%) | 325(20%) | 26% | 13% |
| 1t8GU | 771(28%) | 502(19%) | 365(18%) | 206(7%) | 286(18%) | 18% | 7% |
| 1t8Mi | 276(10%) | 251(9%) | 122(6%) | 185(6%) | 149(9%) | 8% | 28% |
| 1t8In | 164(6%) | 139(5%) | 16(1%) | 8(1%) | 65(4%) | 3% | 6% |
| 1t8De | 349(13%) | 284(11%) | 104(5%) | 198(6%) | 122(8%) | 9% | 31% |
